# Amyloid Precursor Protein and Proinflammatory Changes Are Regulated in Brain and Adipose Tissue in a Murine Model of High Fat Diet-Induced Obesity

**DOI:** 10.1371/journal.pone.0030378

**Published:** 2012-01-19

**Authors:** Kendra L. Puig, Angela M. Floden, Ramchandra Adhikari, Mikhail Y. Golovko, Colin K. Combs

**Affiliations:** Department of Pharmacology, Physiology and Therapeutics, University of North Dakota School of Medicine and Health Sciences, Grand Forks, North Dakota, United States of America; Boston University School of Medicine, United States of America

## Abstract

**Background:**

Middle age obesity is recognized as a risk factor for Alzheimer's disease (AD) although a mechanistic linkage remains unclear. Based upon the fact that obese adipose tissue and AD brains are both areas of proinflammatory change, a possible common event is chronic inflammation. Since an autosomal dominant form of AD is associated with mutations in the gene coding for the ubiquitously expressed transmembrane protein, amyloid precursor protein (APP) and recent evidence demonstrates increased APP levels in adipose tissue during obesity it is feasible that APP serves some function in both disease conditions.

**Methodology/Principal Findings:**

To determine whether diet-induced obesity produced proinflammatory changes and altered APP expression in brain versus adipose tissue, 6 week old C57BL6/J mice were maintained on a control or high fat diet for 22 weeks. Protein levels and cell-specific APP expression along with markers of inflammation and immune cell activation were compared between hippocampus, abdominal subcutaneous fat and visceral pericardial fat. APP stimulation-dependent changes in macrophage and adipocyte culture phenotype were examined for comparison to the *in vivo* changes.

**Conclusions/Significance:**

Adipose tissue and brain from high fat diet fed animals demonstrated increased TNF-α and microglial and macrophage activation. Both brains and adipose tissue also had elevated APP levels localizing to neurons and macrophage/adipocytes, respectively. APP agonist antibody stimulation of macrophage cultures increased specific cytokine secretion with no obvious effects on adipocyte culture phenotype. These data support the hypothesis that high fat diet-dependent obesity results in concomitant pro-inflammatory changes in brain and adipose tissue that is characterized, in part, by increased levels of APP that may be contributing specifically to inflammatory changes that occur.

## Introduction

Obesity, particularly in mid-life, is an increased risk factor for AD independent of other conditions [1], [2], [3], [4], [5], [6], [7]. Particular saturated versus unsaturated fat ingestion at midlife also increases the risk of developing AD [8], [9]. In addition, metabolic syndrome and diabetes, often comorbid with obesity, are factors of increased risk for AD in some [6], [7], [10], [11] but not all studies [12]. Interestingly, late life obesity and metabolic syndrome are either not risk factors or actually decrease the risk of AD in several studies [3], [13], [14]. Others have reported that obesity itself is associated with poorer cognitive performance in humans [15], [16], [17] as well as decreased brain volumes [18] independent of age or disease. In spite of this abundance of correlational data, a particular mechanism linking the pathophysiology of obesity to the brain changes of AD remains unclear.

One possibility of linking the conditions focuses on the biology of amyloid precursor protein, APP. It is expressed in the brain primarily by neurons [19] where it can be metabolized to Aβ1-40 and 1-42 peptides which aggregate to form amyloid plaques characteristic of AD [20]. Moreover, mutations in the gene coding for APP [21] or its protease presenilins [22], [23], [24] are responsible for a rare autosomal dominant form of disease. Therefore, APP and its proteolytic fragments are likely to play a central role in the pathophysiology of AD.

Recent data suggests that APP expression or function may also be involved in the pathophysiology of obesity. It is known that adipose tissue [25], [26], [27] and adipocyte cell lines [27] express APP. More importantly, adipose APP and Aβ1-40 plasma levels increase in obese individuals [25], [26] and plasma Aβ1-42 and 1-40 levels correlate with increased body fat in humans [28], [29]. Rodent studies have examined the brain in a variety of diet-induced obesity paradigms confirming that brain changes leading to increased Aβ levels occur in both AD transgenic [30], [31] and wild type mice [32]. These findings indicate that changes in APP expression or function may be coordinated across diverse tissue types.

In this study a high fat diet-induced model of obesity was used with C57BL6/J mice to determine whether changes in APP expression occurred similarly in brain versus visceral and subcutaneous fat depots in correlation with simultaneous proinflammatory changes in each tissue.

## Results

### High fat diet feeding increased brain levels of APP and multiple pro-inflammatory proteins compared to control diet fed mice

**Figure 1:**
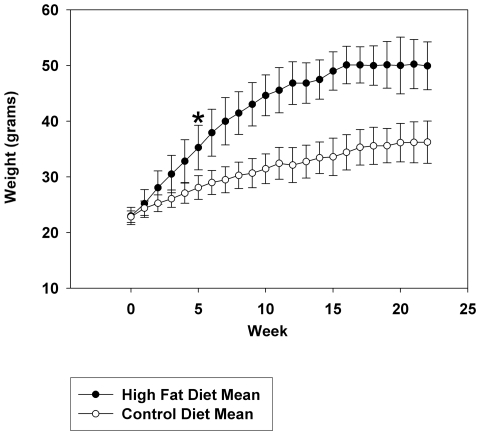
Average weight gain per week for mice fed a high fat versus control diet. C57BL6/J mice at 6 weeks of age and weight matched were fed, *ad libitum*, a control (5.5% fat/weight) or high fat (21.2% fat/weight) diet for 22 weeks. 12 animals in each group were weighed weekly and mean (+/−SD) weight gain per group was graphed versus time. *p = 0.001.

**Figure 2:**
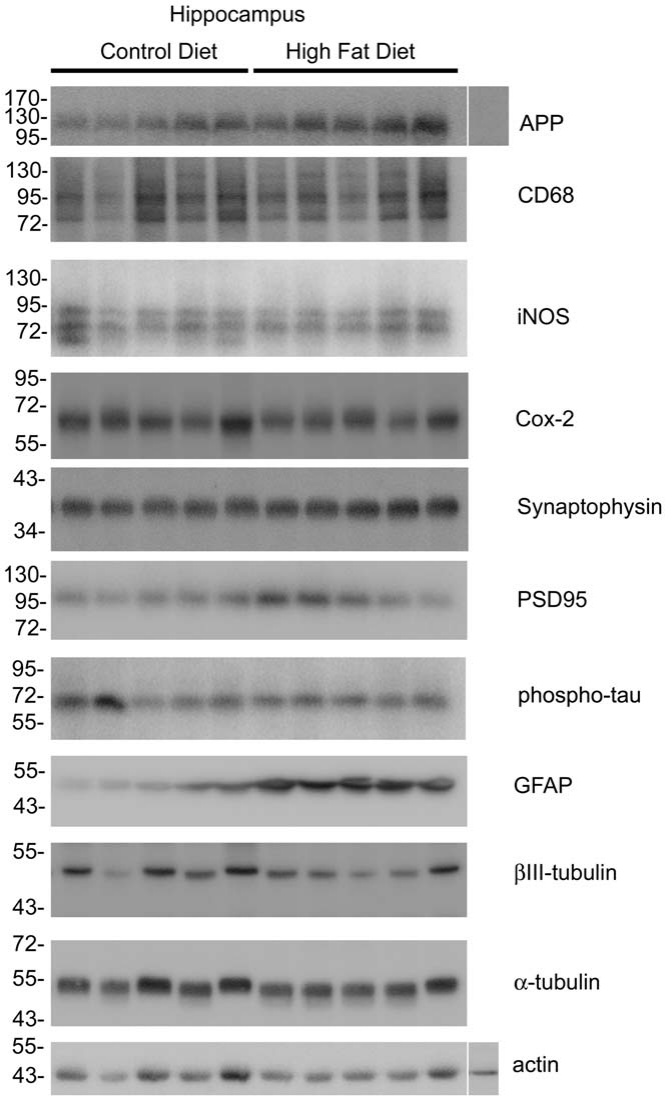
APP and GFAP protein levels were increased in hippocampi of high fat versus control diet fed mice. C57BL6/J mice at 6 weeks of age and weight matched were fed, *ad libitum*, a control (5.5% fat/weight) or high fat (21.2% fat/weight) diet for 22 weeks. Hippocampus samples were collected from 5 animals for each diet. Comparable age hippocampi from APP^-/-^ mice were collected as negative controls for antibody specificity. The tissue was lysed, resolved by 10–15% SDS-PAGE and Western blotted using anti-synaptophysin, PSD95, APP, iNOS, Cox-2, GFAP, phospho-tau (PHF-1), CD68, βIII tubulin (neuronal loading control), α-tubulin and actin (general loading control) antibodies. Arrowheads indicate bands of interest when nonspecific bands are present. Antibody binding was visualized by chemiluminescence. Blots from all animals in each diet are shown.

**Figure 3:**
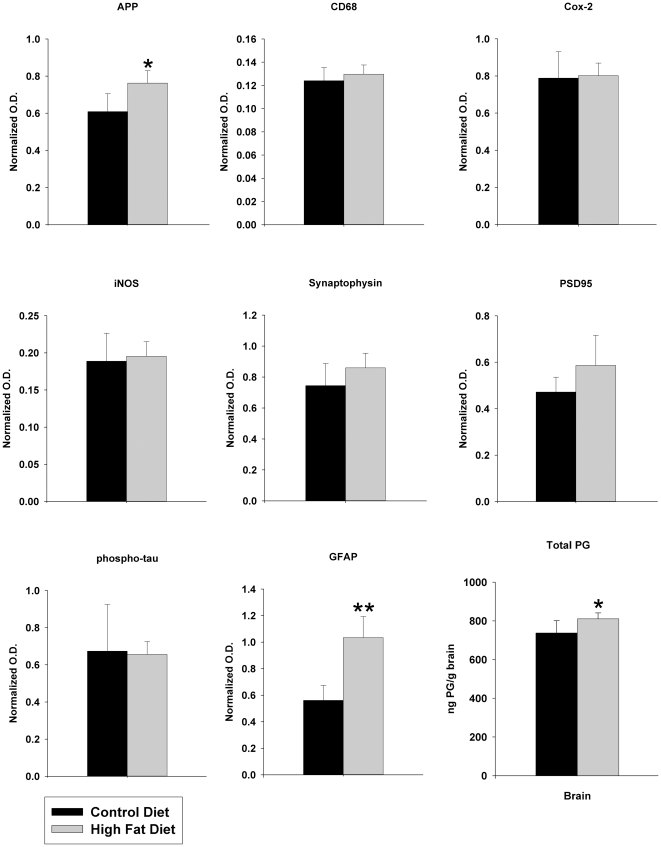
APP and GFAP protein and total prostaglandin levels were increased in the hippocampi of high fat versus control diet fed mice. Optical densities of the Western blotted hippocampal proteins (APP, CD68, iNOS, Cox-2, GFAP, synaptophysin, PSD95, and phospho-tau) from the control and high fat diet Western blots were normalized against their respective averaged O.D. values of combined α-tubulin + βIII tubulin + actin loading controls (+/−SD) from 5 animals for each diet. Total brain PG levels were quantitated as a sum of PGE_2_, PGD_2_, *6-keto*PGF_1α_, PGF_2α_, and thromboxane B_2_ in each diet group. *p<0.05 and **p<0.01.

In order to establish the system for comparing changes in adipose tissue to brain, a standard high fat diet feeding paradigm was used. 24 six week old weight matched male C57BL6/J mice were placed on either a 21.2% by weight high fat diet or a 5.5% by weight control diet, *ad libitum,* beginning at six weeks of age (Table 1). Twelve animals in each group were weighed weekly for 22 weeks and mean (+/−SD) weight gain per group was graphed versus time (Fig. 1). By week five the high fat diet fed mice demonstrated a statistically significant increase in weight gain over the control diet fed mice (Fig. 1). After 22 weeks, the high fat diet fed mice had on average a 217% total weight gain, whereas control diet fed mice had on average only a 158% total weight gain. To examine whether proinflammatory or degenerative changes were occurring in the brains of high fat diet fed animals, Western blot analysis of hippocampi from both groups was performed. As expected, high fat diet fed mice demonstrated a significant increase in expression of APP when compared to control diet fed mice (Figs. 2, 3). This did not correlate with any change in protein levels of the postsynaptic protein marker, PSD95, or the presynaptic marker, synaptophysin (Figs. 2, 3). However, there was a significant increase in astrocyte GFAP protein levels, but no change in microglia CD68 protein levels associated with the high fat diet fed mice. Interestingly, two markers of inflammatory change, iNOS and Cox-2 were not altered in hippocampi of high fat diet versus control diet fed animals (Figs. 2, 3). Although Cox-2 protein levels were not altered, we further examined enzyme activity and total brain prostaglandin (PG) levels (a sum of PGE_2_, PGD_2_, *6-keto*PGF_1α_, PGF_2α_, and thromboxane B_2_) were quantitated from animals in each diet group. Interestingly, high fat diet fed animals demonstrated a significant increase in brain prostaglandin levels compared to control fed animals indicating elevated arachidonic acid metabolism in spite of no significant change in protein levels of Cox-2 (Fig. 3). Additionally, high fat diet feeding did not significantly alter phosphorylation levels of tau protein [33] as assessed using the PHF-1 antibody (Figs. 2, 3). These data demonstrate that high fat diet feeding stimulates an increase in APP protein levels in the brain which correlates with an increased level of gliosis and elevated prostaglandin levels. This supports the notion that the chronic inflammatory changes associated with peripheral tissue during diet-induced obesity extend into the brain. Moreover, these changes are consistent to some degree with those that are observed during Alzheimer's disease.

**Table 1:** Commercial diet formulations for control and high fat diet.

	Control Diet	High Fat Diet
**protein (% by weight)**	22.0	17.3
**carbohydrate (% by weight)**	40.6	48.5
**fat (% by weight)**	5.5	21.2
**energy density (Kcal/g)**	3.0	4.5

### Proinflammatory protein immunoreactivity increased in neurons in brains from high fat diet versus control fed mice

**Figure 4:**
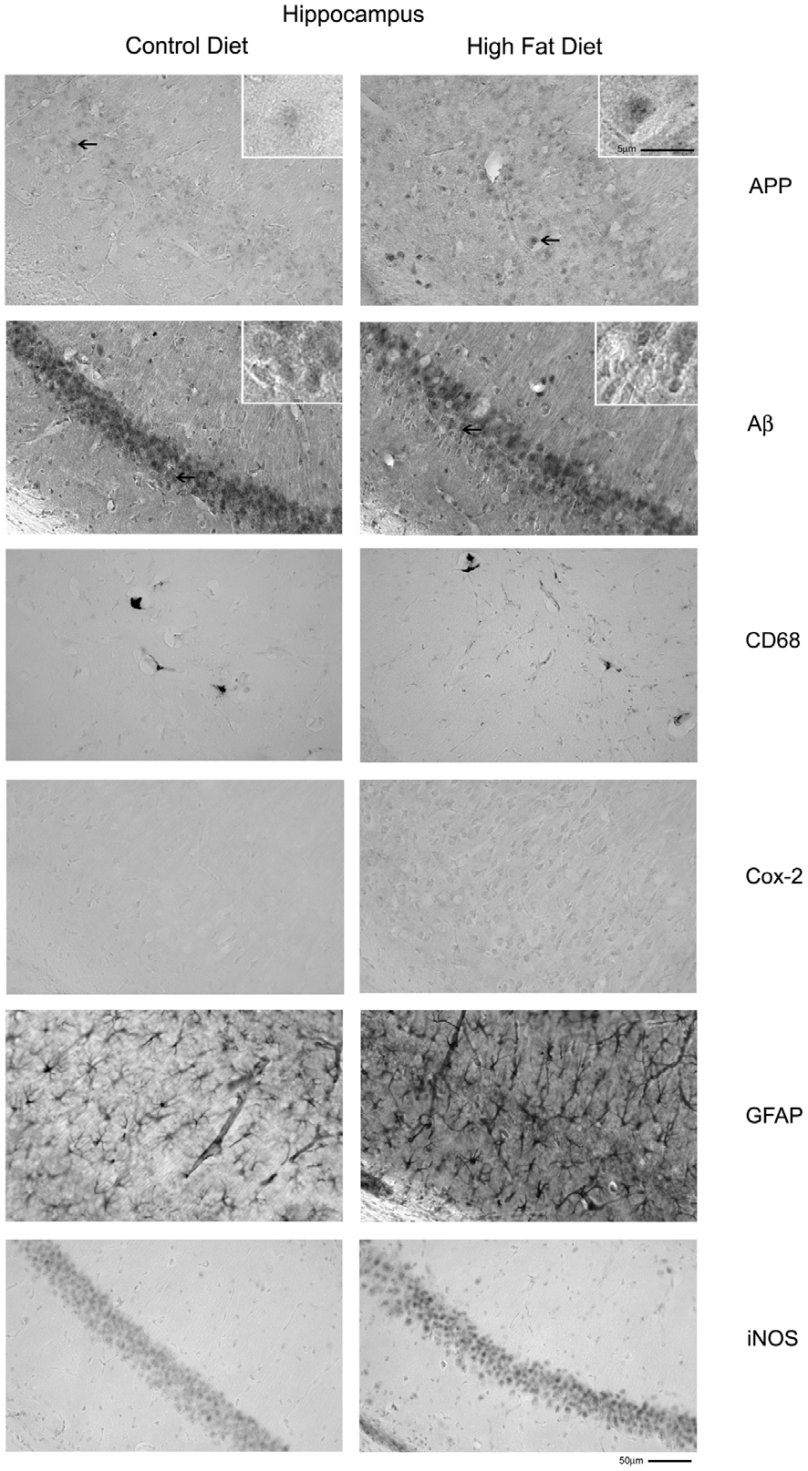
Hippocampi of high fat diet fed mice demonstrated microglial (CD68), astrocytic (GFAP), and neuronal (Cox-2, APP and iNOS) immunoreactivity versus control diet mice. C57BL6/J mice at 6 weeks of age and weight matched were fed, *ad libitum*, a control (5.5% fat/weight) or high fat (21.2% fat/weight) diet for 22 weeks. Tissue samples were collected, fixed in 4% paraformaldehyde, serially sectioned, and immunostained. Tissue sections were immunostained using anti-APP, Aβ, GFAP, CD68, Cox-2, and iNOS antibodies and antibody binding was visualized using Vector VIP as the chromagen. Arrows indicate the location of APP immunoreactivity shown as an enlarged inset in upper right corner of each panel. Representative images from 12 animals per condition are shown.

Although the Western analysis demonstrated significant protein changes in high fat diet fed mice compared to controls, the cellular identity of those proteins was unknown. Understanding the cellular contribution was of particular interest since APP is expressed by several cells in the brain. In order to determine which cells may be responsible for the changes in protein levels, immunohistochemistry was performed from the collected hippocampi. In agreement with the fact that the majority of APP is expressed by neurons in the brain [34] both high fat and control diet fed mice displayed neuronal immunoreactivity for APP with a diet-induced increase in immunoreactivity in the high fat diet fed mice (Fig. 4). To examine whether APP was increasingly processed or deposited as the proteolytic fragment, beta amyloid (Aβ), brain sections were also immunostained using an anti-mouse Aβ antibody. However, there was no obvious change in intensity or distribution of Aβ immunoreactivity in the different diet fed animals (Fig. 4). Cox-2, iNOS, and CD68 histologic analysis demonstrated what appeared to be elevated immunoreactivity for each (Fig. 4) although this did not reach the level of statistical significance via Western blot analysis (Fig. 3). Interestingly, the immunoreactivity for both Cox-2 and iNOS in high fat diet fed animals appeared within neurons as opposed to glia (Fig. 4). Although, the difference in immunoreactivity for reactive microglia (CD68) was modest, there was a clear increase in astrocyte GFAP immunoreactivity in the high fat diet fed animals compared to controls demonstrating that at least an astrogliosis was occurring (Fig. 4). These data demonstrated that high fat diet feeding induced a reactive gliosis in the brain. However, since the Western blot analysis detected increased protein levels of APP within neurons, the proinflammatory changes were not limited to any particular cell type in the brain but instead appeared to include a multi-cellular response.

**Figure 5:**
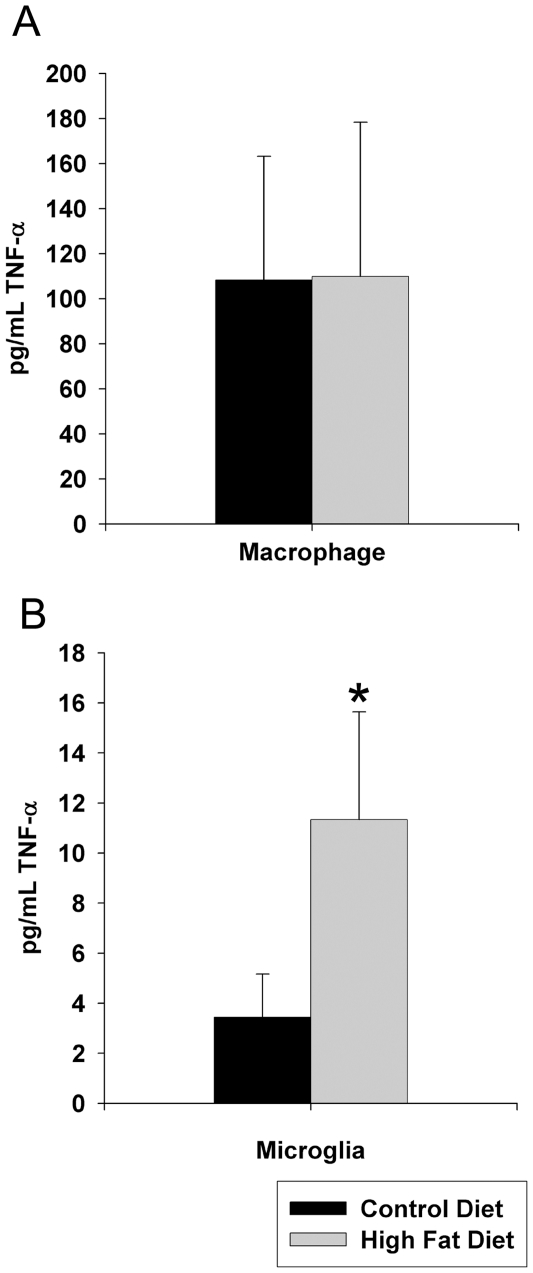
Microglia but not peritoneal macrophage demonstrated increased TNF-α secretion. C57BL6/J mice at 6 weeks of age and weight matched were fed, *ad libitum*, a control (5.5% fat/weight) or high fat (21.2% fat/weight) diet for 22 weeks. Microglia were isolated from brains for comparison to non-elicited peritoneal macrophage. Isolated microglia or macrophage from control and high fat diet fed mice were plated overnight in serum free DMEM/F12 media and secreted TNF-α levels were quantified by commercial ELISA. Data is the average (+/−SD) of 12 animals/condition. *p<0.05.

In spite of the fact that microglia and astrocytes demonstrated immunoreactivity for typical activation markers, CD68 and GFAP respectively, this was not necessarily a reflection of a contribution to any proinflammatory changes that were occurring. As a means of assessing this, microglia were further examined based upon our prior experience with isolating these cells from adult animals [35]. For comparison, microglial secreted TNF-α levels were compared to values derived from resting peritoneal macrophage as a positive control. In general microglia isolated from the 22 week old control fed animals secreted far less TNF-α when compared to their peripheral cell counterparts (Fig. 5). However, microglia from high fat diet fed mice secreted significantly more TNF-α than the control diet fed animals (Fig. 5). This was entirely consistent with the subtle increase in CD68 immunoreactivity observed even though quantified Western blot analysis revealed no significant difference in CD68 protein levels in the brains of high fat diet fed mice compared to controls. Taken together, these data support the idea that proinflammatory changes occur in brains of high fat diet fed animals. These involved not only neuronal upregulation of proteins but also increased cytokine secretion from reactive glia.

### High fat diet feeding increased APP and TNF-α protein levels compared to control diet fed mice in both subcutaneous and visceral fat depots

**Figure 6:**
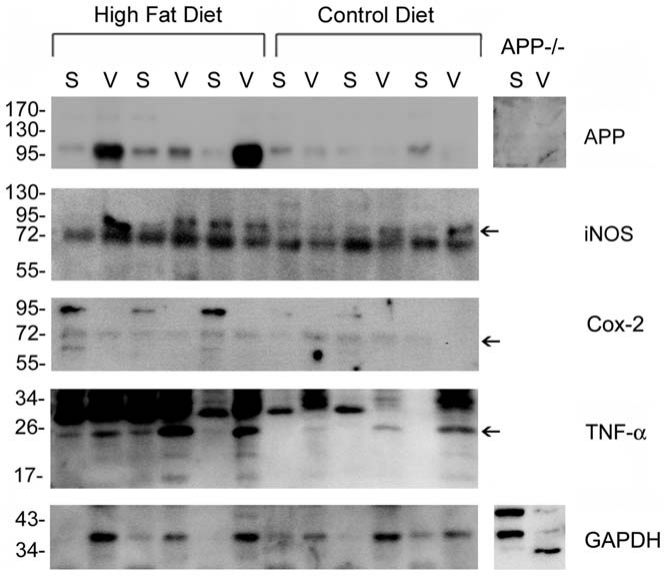
APP and TNF-α levels increased in visceral and subcutaneous fat depots in high fat diet fed animals versus controls. C57BL6/J mice at 6 weeks of age and weight matched were fed, *ad libitum*, a control (5.5% fat/weight) or high fat (21.2% fat/weight) diet for 22 weeks. Pericardial visceral (v) and abdominal subcutaneous (s) fat were collected from 12 animals per group. The tissue was lysed, resolved by 10-15% SDS-PAGE and Western blotted using anti-APP, iNOS, Cox-2, TNF-α, and GAPDH (loading control) antibodies. Arrowheads indicate bands of interest when nonspecific bands are present. Antibody binding was visualized by chemiluminescence. A representative blot of three animals per condition from a total of 12/condition analyzed is shown.

**Figure 7:**
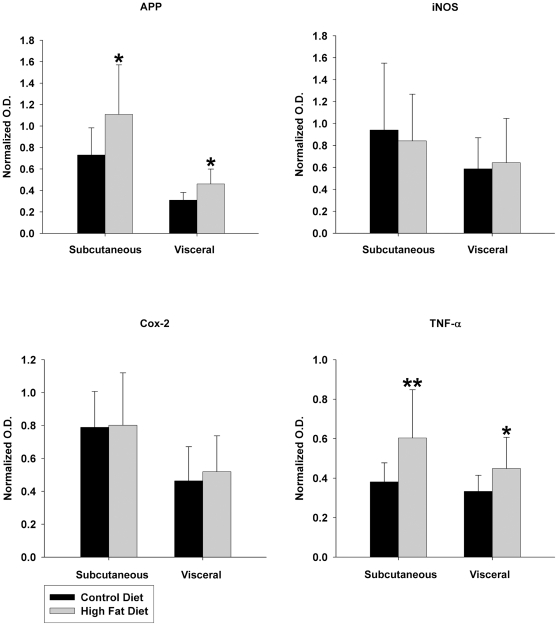
APP and TNF-α protein levels increased in subcutaneous abdominal and visceral pericardial fat in high versus control diet fed mice. Optical densities of the adipose protein Western blots from the control and high fat diet subcutaneous and visceral samples were normalized against their respective GAPDH loading controls and averaged (+/− SD) from 12 animals per each condition. *p<0.05, **p<0.01.

Based upon the changes observed in the brain, adipose tissue was next examined to determine whether similar changes in proinflammatory protein expression occurred in the periphery. Because visceral and subcutaneous fat depots can have altered protein expression changes during diet-induced obesity [36], [37], [38], [39] both types of adipose reservoirs were assessed. To begin comparing protein changes between brain and adipose tissue, Western blot analysis was again performed. Subcutaneous abdominal fat and visceral pericardial fat were examined as representative samples of two distinct fat depots. Precisely as observed in the brain, high fat diet fed mice demonstrated a significant increase in APP protein levels in both fat depots over control diet fed mice (Figs. 6, 7). To again assess if there was a proinflammatory change, the two proinflammatory protein markers quantified from brain, iNOS and Cox-2, were next examined in the fat depots. Consistently, the diets demonstrated no difference in either iNOS or Cox-2 protein levels in either type of adipose tissue (Figs. 6, 7). However, based upon the fact that microglial-secreted TNF-α levels were increased in high fat diet fed mice and TNF-α elevations are a well characterized change in adipose tissue from obese individuals [40], [41] or animals [42], we next quantified TNF-α protein levels. Similar to the changes observed from brain microglia, both visceral and subcutaneous fat depots demonstrated increased TNF-α levels compared to pair fed controls (Figs. 6, 7). These data demonstrate that although there were no significant differences between visceral and subcutaneous fat depots, the overall proinflammatory changes were consistent between adipose tissue and brain during high fat diet feeding. In particular, the proinflammatory proteins, Cox-2 and iNOS, were not significantly increased in either the brain or adipose tissue in spite of the observed elevation in TNF-α from both tissue types. Perhaps the most interesting observation was that APP levels increased in both brain and adipose tissue. This demonstrated that a coordinated, increased expression of APP occurred in the brain and adipose tissue upon diet-induced obesity along with acquisition of proinflammatory tissue phenotypes.

### Adipose tissue APP immunoreactivity from high fat diet fed animals localized to adipocytes and macrophage

**Figure 8:**
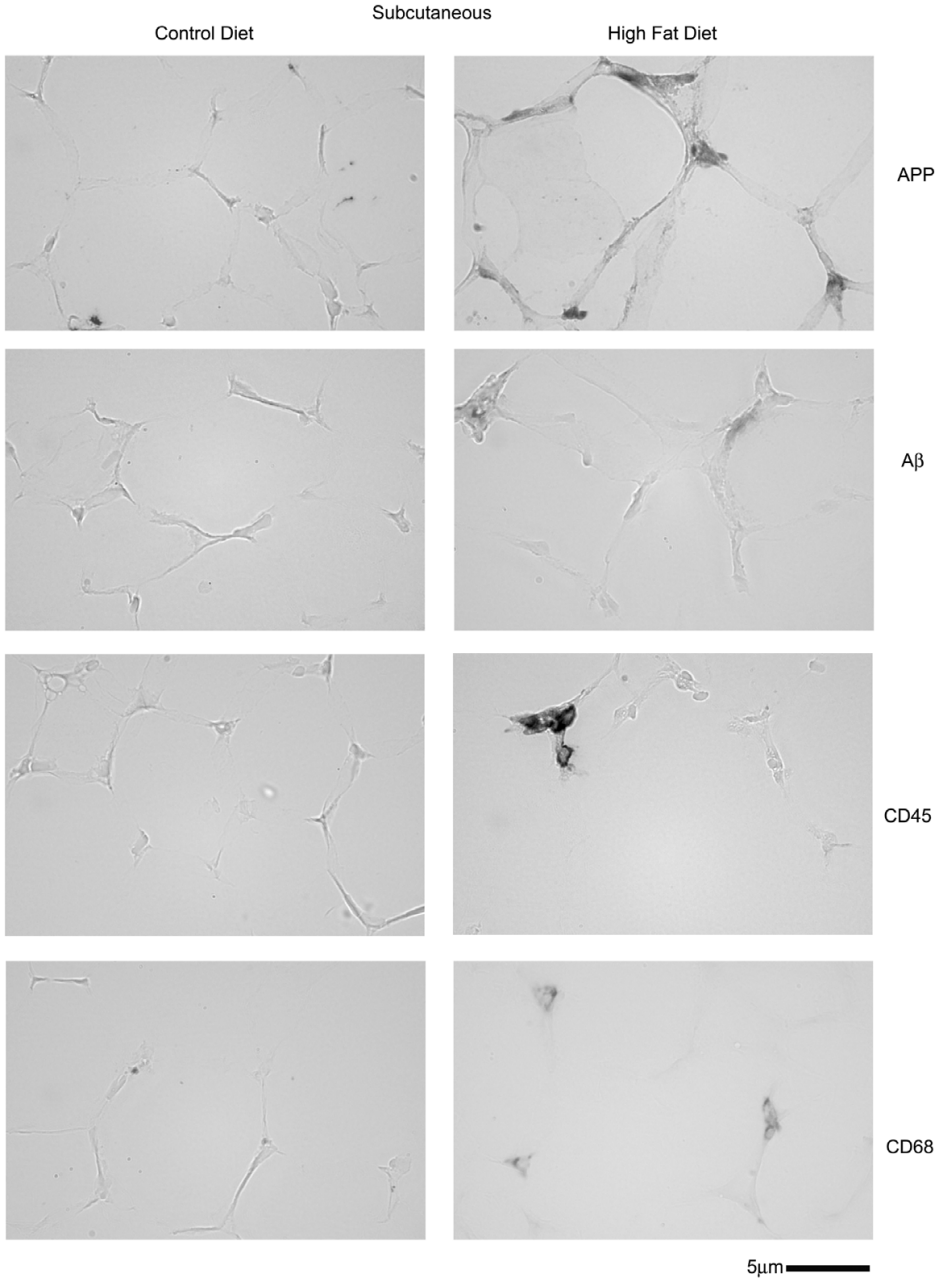
APP, CD45, and CD68 immunoreactivity increased in the subcutaneous (abdominal) fat from high fat versus control diet fed mice with no robust change in Aβ immunoreactivity. C57BL6/J mice at 6 weeks of age and weight matched were fed, *ad libitum*, a control (5.5% fat/weight) or high fat (21.2% fat/weight) diet for 22 weeks. Subcutaneous abdominal adipose tissue was collected, immersion fixed in 4% paraformaldehyde, sectioned, and immunostained using anti-APP, Aβ, CD45 and CD68 antibodies and antibody binding visualized using Vector VIP as the chromogen. Representative images from 12 animals per condition are shown.

**Figure 9:**
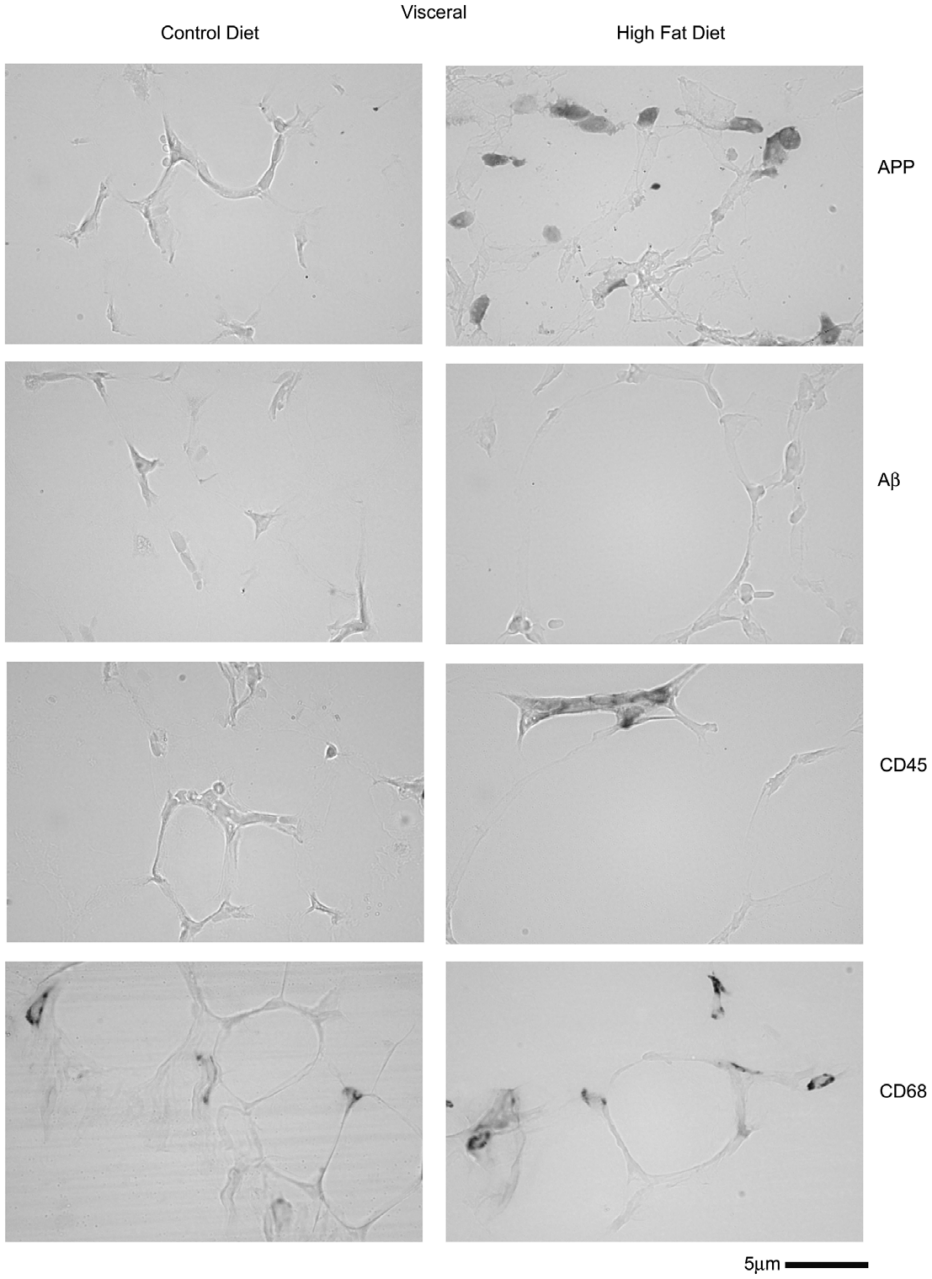
APP, CD45, and CD68 immunoreactivity increased in the visceral (pericardial) fat from high fat versus control diet fed mice with no robust change in Aβ immunoreactivity. C57BL6/J mice at 6 weeks of age and weight matched were fed, *ad libitum*, a control (5.5% fat/weight) or high fat (21.2% fat/weight) diet for 22 weeks. Visceral pericardial adipose tissue was collected, immersion fixed, sectioned, and immunostained using anti-APP, Aβ, CD45 and CD68 antibodies and antibody binding visualized using Vector VIP as the chromogen. Representative images from 12 animals per condition are shown.

**Figure 10:**
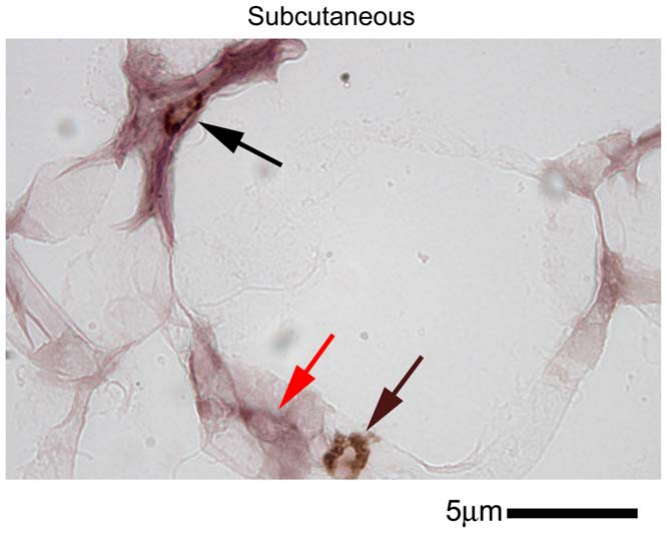
APP and CD68 immunoreactivity co-localized in subcutaneous (abdominal) and visceral (heart) fat from high fat diet fed mice. C57BL6/J mice at 6 weeks of age and weight matched were fed, *ad libitum*, a control (5.5% fat/weight) or high fat (21.2% fat/weight) diet for 22 weeks. Subcutaneous abdominal and visceral pericardial adipose tissue samples were collected, immersion fixed, serially sectioned and immunostained using anti-CD68 antibody and binding visualized using DAB as the chromogen. For double-labeling, tissue sections were stripped using 0.2N HCl and subsequently immunostained using anti-APP antibody and binding visualized using Vector VIP as the chromogen. A representative image from 12 animals per condition is shown. The brown arrow indicates CD68 immunoreactivity, the red arrow indicates APP immunoreactivity and the black arrow indicates double-label of CD68 and APP antibody binding.

Although brain changes in APP were determined to be neuronally localized it was not likely that the increased APP levels in adipose tissue were also neuronal. Therefore, to identify the cellular origin of the increased APP levels in high fat diet fed adipose tissue immunohistochemistry was again performed. In both subcutaneous (Fig. 8) and visceral (Fig. 9) adipose tissue increased APP immunoreactivity was observed in high fat diet fed versus control diet fed animals. Similar to the observations found during brain analysis, immunostaining of the two different adipose depots with anti-mouse Aβ antibody did not demonstrate a robust increase in immunoreactivity in high fat diet fed animals nor any plaque-type patterns (Figs. 8, 9). Although prior work has demonstrated increased adipocyte APP immunoreactivity in samples from obese humans, it was possible that the increased APP immunoreactivity observed in our animal paradigm was also localized to macrophage. Our prior work has shown that monocytic lineage cells express APP and levels increase upon differentiation and activation [43], [44], [45]. Moreover, a variety of studies have demonstrated that increased macrophage infiltration into adipose tissue occurs during diet-induced obesity [46], [47], [48]. Interestingly, both subcutaneous and visceral adipose tissue demonstrated increased immunoreactivity for CD68 to identify macrophage (Figs. 8, 9). Importantly, this increase in CD68 positive adipose tissue macrophage correlated precisely with the slight increase in CD68 positive microglia observed in the brains of high fat diet fed mice compared to controls (Fig. 4). Double labeling immunohistochemistry was performed to determine whether a portion of the increased APP immunoreactivity observed was within adipose tissue infiltrated macrophage. In either type of adipose tissue a population of the APP immunoreactive cells also co-localized with CD68 immunoreactivity indicating that both adipocytes and macrophage may be responsible for the upregulation of APP observed in adipose tissue of high fat diet fed animals (Fig. 10). Indeed, visceral adipose tissue demonstrated nearly complete co-localization of APP with CD68 immunoreactivity. These results confirm that increased APP levels observed in adipose tissue from high fat diet fed animals is multi-cellular with a significant portion likely from the increasing numbers of tissue infiltrating macrophage. This correlates with the increased levels of brain APP observed in high fat diet fed mice as well as the increased gliosis that occurs.

### The APP agonist antibody, 22C11, increased macrophage cytokine production but had no effect on viability, lipid storage/accumulation, or TNF-α secretion in adipocytes

**Figure 11:**
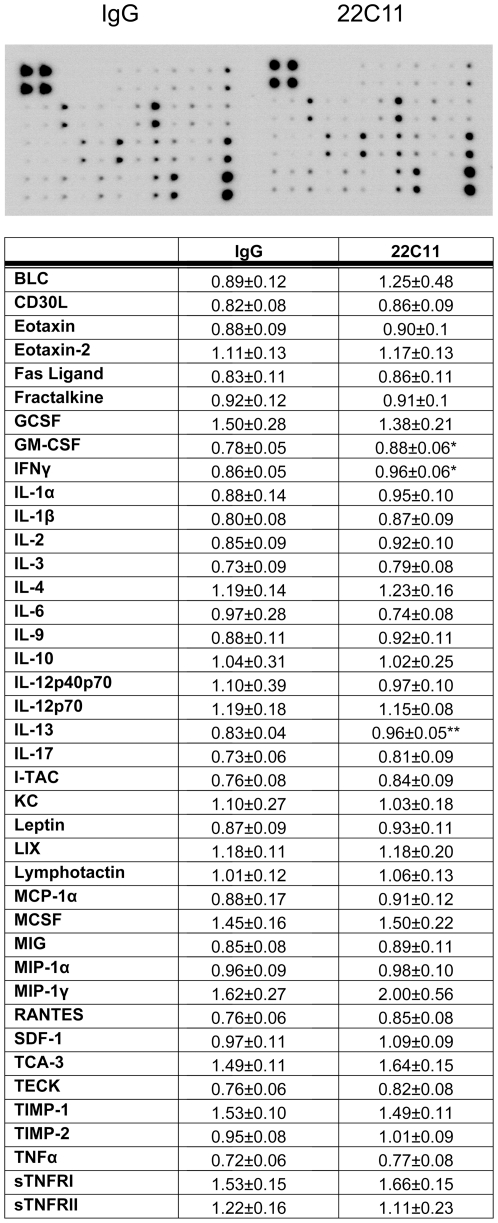
Secreted levels of GM-CSF, IFNγ, and IL-13 increased in media from peritoneal macrophage stimulated with APP agonist antibody, 22C11, compared to IgG_1_ isotype control. Non-elicited peritoneal macrophage were stimulated with 1 µg/mL IgG_1_ or 1 µg/mL 22C11 APP agonist antibody overnight and the media was removed and used according in a commercial antibody-based 40 cytokine antibody array. A representative dot blot per each condition is shown. The optical densities of individual cytokine detection spots were normalized against their respective positive controls per blot and averaged (+/−SD) and graphed from 5 animals in each group. Data were analyzed via unpaired two-tailed t-test. *p<0.05, **p<0.01.

To begin examining whether increased expression of APP had any role in altering the phenotype of macrophage or adipocytes, primary murine cultures were generated from non-elicited peritoneal macrophage and subcutaneous adipocytes and then stimulated using an agonist antibody for APP [44]. We first stimulated peritoneal macrophage with 1 µg/mL IgG_1_ (isotype control) or 22C11 (APP agonist antibody) and measured cytokine secretion (Fig. 11). The APP agonist, 22C11, stimulated a significant increase in secretion of granulocyte-macrophage colony stimulating factor (GM-CSF) which reportedly increases the production of macrophages [49]. This was consistent with the increase in immunoreactivity for CD68 (macrophages) in both adipose tissue depots during high fat diet-induced obesity. Stimulation with 22C11 also significantly increased secreted levels of IFNγ, a macrophage-activating factor, that plays a critical role in immunostimulatory and immunomodulatory effects [50], [51]. In contrast, 22C11 stimulation significantly increased secreted IL-13 levels which is reportedly responsible for down-regulation of macrophage activity and thereby inhibits the production of pro-inflammatory cytokines and chemokines [52]. Although these findings do not demonstrate precisely what secretory changes APP-overexpressing macrophage may be exhibiting *in situ* during diet-induced obesity, they do provide clear evidence that APP stimulated changes in macrophage phenotype are complex with alterations in both proinflammatory and anti-inflammatory secretion that will need to be further resolved *in vivo* in the diet-induced obesity model.

**Figure 12:**
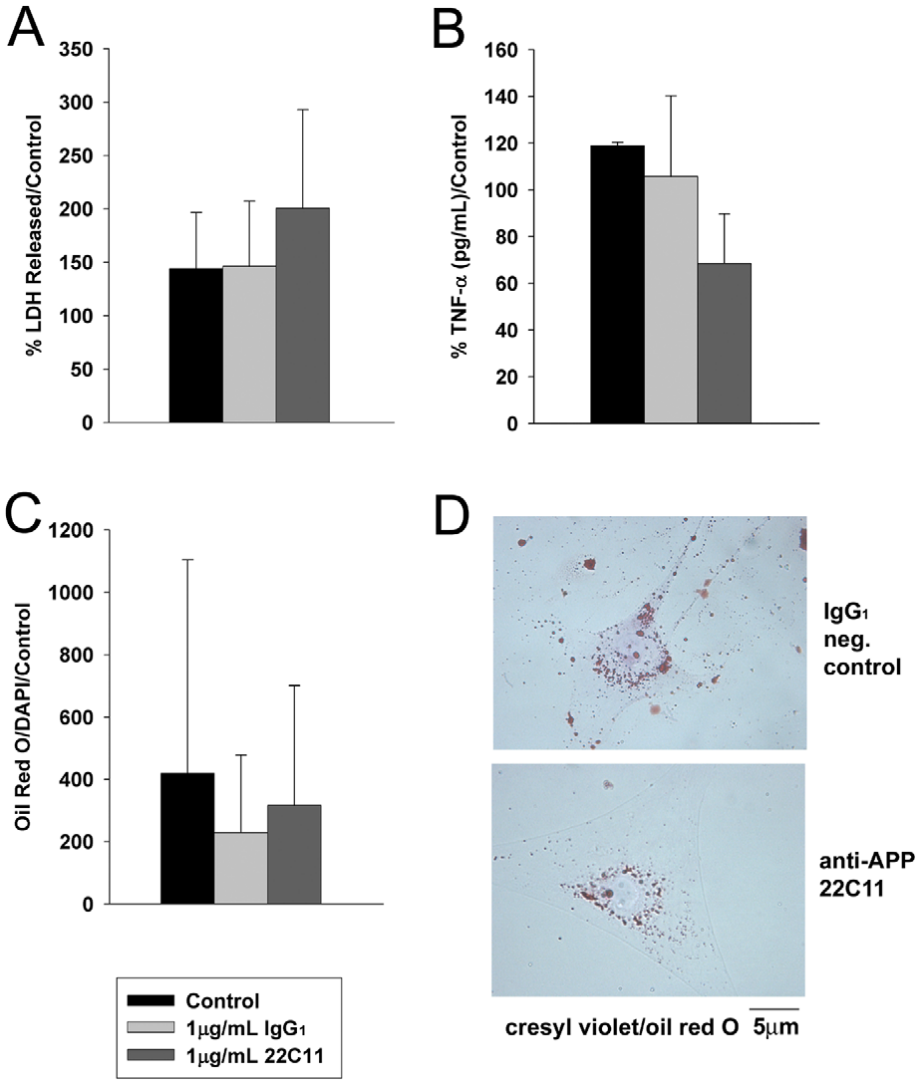
APP activation with agonist antibody did not alter cytokine secretion, viability, or Oil Red O staining of primary murine abdominal subcutaneous adipocytes. Adipocytes were harvested from subcutaneous abdominal fat from C57BL6/J mice and cultured in DMEM/F12 with serum and antibiotics for one week. Cells were then placed into serum free DMEM/F12 and unstimulated (control) or stimulated for 24 hr with 1 µg/mL IgG_1_ (isotype control) or 1µg/mL 22C11 (APP agonist antibody). After 24 hours (**A**) Secreted LDH released into the media normalized against cellular LDH was quantified to assess changes in cell viability upon APP stimulation. (**B**) Changes in secreted TNFα levels were also quantified from the media as a result of APP stimulation. (**C**) Cells were also fixed, stained with Oil Red O and DAPI counterstain and changes in Oil Red O absorbance (510 nm) normalized to DAPI absorbance (358/461 nm) were quantified via plate reader. (**D**) Representative images of Oil Red O and cresyl violet counterstained adipocytes are shown following 24 hr IgG_1_ and 22C11 stimulation. Data are shown as mean (+/−SD) and are representative of three independent experiments. Data were analyzed via a one-way ANOVA.

To assess any effect of APP on adipocyte phenotype, we next stimulated primary murine abdominal subcutaneous fat derived-adipocytes with 1 µg/mL IgG_1_ or 22C11 antibodies. Unlike the macrophage studies, agonist antibody stimulation of adipocytes did not produce any obvious change in phenotype. There was no significant toxicity of the 22C11 or IgG_1_ stimulated adipocytes as assessed by LDH release (Fig. 12A). Since TNFα levels were increased under diet-induced obesity conditions in both adipose tissue depots, TNFα secretion was measured from APP stimulated adipocytes (Fig. 12B). However, the APP agonist, 22C11, did not stimulate a significant change in TNFα secretion. To assess a differentiative phenotype, stimulated adipocytes were stained and quantified using Oil Red O (Fig. 12C and 12D) to examine lipid storage/accumulation. APP stimulation with 22C11 produced no changes in Oil Red O staining. Although a minor subset of all the possible changes in adipocyte phenotype was examined, the data thus far indicates that viability, proinflammatory secretion, and differentiation state are not affected by APP signaling. This suggests that further investigation is needed to fully understand the role of APP in adipocyte biology both basally and during instances of increased APP expression such as that which occurs during diet-induced obesity.

## Discussion

We demonstrate that a high fat diet feeding paradigm in C57BL6/J mice drives a concomitant increase in protein levels of APP in brain and visceral and subcutaneous adipose tissue correlating with proinflammatory changes in a cell specific fashion. APP protein levels increased within primarily neurons in the brain and macrophage and adipocytes in adipose tissue of high fat diet fed mice. This correlated with reactive gliosis and elevated microglial TNFα production. This elevated proinflammatory, neurodegenerative phenotype of the high fat diet fed brain correlated with a similar increase in APP and TNFα levels in high fat diet fed adipose tissue. This supports the idea that a particular function of APP may contribute to the metabolic changes that occur in each tissue during diet-induced obesity. At the very least, the APP changes observed may serve as one of the growing list of bio-markers that identify obesity-related changes.

In particular, we provide a crucial data set of comparison changes in brain versus adipose tissue that was lacking from earlier studies which have focused on a particular organ. Prior work from human subjects has demonstrated increased adipose tissue APP and plasma Aβ levels in obese subjects [25], [26], [27], [28], [53] although comparable brain data was not reported. Similarly, brain but not adipose data from Alzheimer's disease transgenic rodent studies using diet-induced obesity paradigms have indicated that obesity correlates with increased levels of brain Aβ [30], [31], [54] although not necessarily an increase in full length APP as we observed. Our findings are in greater agreement with those of Thirumanagalakudi and colleagues who found using wild type C57BL6/J mice that a high fat/high cholesterol diet induced increased brain changes in APP, Aβ, and diverse proinflammatory proteins [32] although a related study in C57BL6/J mice reported no change in brain APP or Aβ levels [55]. To link these diverse data sets we suggest, that based upon our findings, a high fat feeding paradigm producing a diet-induced obesity in animals expressing APP under the control of its endogenous promoter is sufficient to increase APP expression in both adipose and brain tissue simultaneous with similar proinflammatory changes that occur in each tissue.

It is not clear what the exact stimulus is to increase APP levels in the cell types in our paradigm although we speculate that proinflammatory stimuli are responsible. It is well established that obesity correlates with a host of increased circulating proinflammatory molecules [56], [57], [58], [59] released by both adipocytes and adipose macrophage [58], [60], [61]. Prior work from the 3T3-L1 adipocyte cell line has demonstrated that stimulation with TNF-α is sufficient to increase APP expression [27]. We [43], [44], [45] as well as others [62], [63], [64], [65], [66], [67], [68], [69], [70], [71], [72], [73] have demonstrated that APP expression and plasmalemmal localization in monocytic lineage cells, including macrophage and microglia, increases during proinflammatory or degenerative conditions. Finally, neurons themselves have a well established ability to increase APP expression during diverse degenerative and inflammatory stimulations [74], [75], [76], [77], [78], [79], [80].

It is important to point out that focus of this work was on the particular cellular changes in full-length APP rather than assessment of APP processing to Aβ. However, we did assess potential Aβ generation and deposition in both brain and visceral and subcutaneous adipose tissue via immunostaining. Although the mouse Aβ-specific antibody detected no robust changes in either tissue from control or high-fat diet fed mice it is possible that Aβ production was increased in parallel with the increased APP protein levels observed in high fat diet-fed mice but was simply not detectable via immunostaining. We have not ruled out the possibility that a longer feeding paradigm and more sensitive detection method such as an Aβ ELISA might demonstrate a significant difference in APP processing to increased levels of Aβ between diets. Indeed, prior work has already demonstrated that high fat/cholesterol feeding increases Aβ levels in the murine brain [32]. This indicates, perhaps not surprisingly, that although APP levels were increased in brain and adipose tissue, its processing and perhaps function(s) is unique based upon cell type expression and requires future study. Regardless of what cell type is potentially producing the Aβ in brain and adipose tissue, the peptide in either its oligomeric or fibrillar form has been shown in numerous studies to be a potent stimulus for activating microglia and monocyte/macrophage cells to acquire a reactive phenotype [81], [82], [83], [84], [85], [86], [87], [88]. Again, although processing of APP to Aβ was not the focus of this work, it is intriguing to consider that in addition to proinflammatory stimuli potentially driving increased APP expression, increased Aβ production may act in a feed-forward fashion to increase proinflammatory secretions in brain and adipose tissue by directly stimulating microglia and macrophage, respectively. These data support the idea that limiting inflammatory changes during diet induced weight gain may not only attenuate pathologic events in peripheral organs but also those in the brain. Indeed, it appears that use of non-steroidal anti-inflammatory drugs during mid-life, in particular, may offer some protective benefit against developing AD [89].

Although our study has focused specifically on changes related to diet induced obesity it is difficult not to speculate that the changes observed may be directly relevant to the mechanism of Alzheimer's disease. As already mentioned, mid-life obesity is a well-recognized increased risk factor for developing AD [1], [2], [3], [4], [6], [7] and several rodent studies using transgenic mouse models of AD have demonstrated that diet-induced obesity paradigms increase Aβ levels in the brain [30], [31], [54]. More importantly, caloric restriction of these transgenic models is sufficient to decrease brain Aβ levels and plaque load [90]. It was particularly interesting that microglia isolated from high fat diet fed mice basally secreted elevated levels of TNFα compared to microglia from control diet fed mice. The ability to isolate these cells acutely from adult mouse brains without the confound of prolonged *in vitro* culturing in serum containing conditions allows us to quantify with confidence the basal microglial secretory phenotype in the brain during either diet paradigm. The elevated proinflammatory state suggested by the glia was supported by elevated levels of total prostaglandins in the high-fat diet fed mice. Although we did not attempt to determine effects of APP stimulation on neuronal phenotype in this study it is interesting to speculate that APP-dependent stimulation of neurons may lead directly to increased neuronal prostaglandin production as well as generation of Aβ that may be direct stimuli for the increased microglial TNFα secretion that occurred in high-fat diet fed brains. This APP-dependent mechanism linking generation of these proinflammatory mediators with gliosis would certainly be reasonable to consider during similar degeneration events in AD.

Perhaps even more interesting is the possibility that APP dependent proinflammatory events contribute to the classic inflammatory changes commonly observed in peripheral adipose tissue during diet-induced obesity. For instance, based upon the increased APP levels observed in macrophage and adipocytes, we examined a role for APP in regulating the phenotype of these cells. Although we were unable to determine any phenotype change in adipocyte downstream of APP stimulation, macrophage exhibited a significant increase in secretion of three particular cytokines out of the 40 analyzed that may be relevant to adipose changes observed during high fat diet feeding. APP stimulation increased macrophage secretion of GM-CSF, IFNγ, and IL-13. GM-CSF has a well characterized role in regulating infiltration of macrophage into adipose tissue [91]. An APP-dependent increase in GM-CSF secretion would certainly help to explain some of the observed increased in reactive macrophage in the high fat diet adipose tissue. IFNγ has an increasingly apparent role in regulating not only adipocyte cytokine secretion including TNFα but also insulin resistance and infiltration of T cells into obese adipose tissue [92], [93], [94]. Elevated IL-13 expression is a hallmark of recently defined alternative M2 phenotype macrophage in obese adipose tissue [95]. Although APP stimulation did not alter adipocyte phenotype in our hands a more extensive assessment would likely identify APP-dependent changes in adipocytes relevant to obesity. One interesting possibility is that an APP-APP dependent interaction between adipocytes and macrophage is involved in activating both cell types. That is, APP on macrophage may interact with APP on adipocytes in a complex trans APP-APP interaction as APP can act as a receptor for monocytic lineage cells [43]. It is known that although adipocytes are quite capable of secreting a range of inflammatory molecules [96] that macrophage-adipocyte interaction can potentiate inflammatory changes that can occur during obesity and metabolic disorder [97]. Determination of specific roles for APP in macrophage and adipocyte changes during high fat diet feeding might be addressed in future work through the use of APP knockout mice or cell specific APP deletion or even expression of mutant forms of APP such as those associated with AD.

A final interesting speculation is that if APP and/or Aβ levels increase in adipose tissue during obesity and AD and coordinated expression is tightly regulated between brain and fat then it is not unreasonable to predict that monitoring APP expression and metabolism in adipose tissue could serve as a surrogate for brain with regard to assessing efficacy of particular drug interventions or monitoring disease pathophysiology of obesity or AD. For instance, it is possible that a portion of any generated Aβ in adipose tissue could accumulate as amyloid deposits in either obese or AD individuals. It is well established that adipose tissue can accumulate amyloid proteins, for example, in individuals with rheumatoid arthritis which is often assessed via needle biopsy [98], [99].

## Materials and Methods

### Ethics Statement

The investigation conforms to the National Research Council of the National Academies *Guide for the Care and Use of Laboratory Animals* (8^th^ edition). Animal use was approved by the University of North Dakota IACUC, protocol #1003-1.

### Materials

Anti-β-Amyloid Precursor Protein (APP) antibody was purchased from Invitrogen (Carlsbad, CA, USA). Anti-mouse IgM (goat), anti-rabbit (goat), anti-goat (bovine), anti-rat (goat), and anti-mouse (bovine) horseradish peroxidase-conjugated secondary antibodies, Cox-2, GAPDH, α-tubulin, and β actin antibodies were purchased from Santa Cruz Biotechnology (Santa Cruz, CA, USA). Elite Vectastain ABC Avidin and Biotin, Vector VIP, Vector DAB, biotinylated anti-rabbit, anti-mouse, and anti-rat antibodies were purchased from Vector Laboratories Inc (Burlingame, CA, USA). Synaptophysin and βIII tubulin antibodies were purchased from Chemicon international, Inc (Temecula, CA, USA). CD45 antibody was purchased from BD Biosciences Pharmingen (San Jose, CA, USA). TNF-α antibody was purchased from Abcam Inc (Cambridge, MA, USA). CD68 antibody was purchased from AbD Serotec (Oxford, UK). iNOS antibody was purchased from Alexis Biochemicals (San Diego, CA, USA). GFAP and PSD95 antibody was purchased from Cell Signaling Technology Inc (Danvers, MA, USA). PHF-1 antibody was a gift from Dr. Peter Davies (Albert Einstein College of Medicine, NY). Cox-2 and Prostaglandin (PG) E_2_d4 were purchased from Cayman Chemical (Ann Arbor, MI, USA). β-Amyloid (Aβ), rodent specific polyclonal antibody (SIG-39151) was purchased from Covance (Emeryville, CA, USA).

### Mice

APP^tm1Dbo^/J homozygous (APP^-/-^) mice and wild type (C57BL6/J) mice were purchased from Jackson Laboratory. Mice were provided food and water *ad libitum* and housed in a 12 hour light/dark cycle.

### High Fat vs. Control Diet Feeding

At six weeks of age, 34 male weight matched C57BL6/J wild type mice were placed on either a 21.2% by weight high fat diet (Harlan Teklad TD.88137) or a 5.5% by weight regular fat diet (Harland Teklad 8640), *ad libitum*. 12 animals in each group were weighed each week for 22 weeks.

### Western Blotting

After 22 weeks the animals were perfused with PBS containing CaCl_2_ and brain, hippocampus, visceral (pericardial or heart) fat and subcutaneous (abdominal or stomach) fat were collected and lysed using ice cold RIPA buffer (20 mM Tris, pH 7.4, 150 mM NaCl, 1 mM Na_3_VO_4_, 10 mM NaF, 1 mM EDTA, 1 mM EGTA, 0.2 mM phenylmethylsulfonyl fluoride, 1% Triton, 0.1% SDS, and 0.5% deoxycholate) with protease inhibitors (AEBSF 1 mM, Aprotinin 0.8 µM, Leupeptin 21 µM, Bestatin 36 µM, Pepstatin A 15 µM, E-64 14 µM). To remove insoluble material cell lysates were sonicated and centrifuged (14,000 rpm, 4°C, 10 min). The Bradford method [100] was used to quantify protein concentrations. Proteins were resolved by 10% SDS-PAGE and transferred to polyvinylidene difluoride membranes for Western blotting using anti-APP, iNOS, Cox-2, synaptophysin, PSD95, phospho-tau (PHF-1), TNF-α, GFAP, CD68, βIII tubulin (loading control), α-tubulin (loading control), GAPDH (loading control). Antibody binding was detected with enhanced chemiluminescence (GE Healthcare, Piscataway, NJ, USA). In some instances, antibodies were stripped from blots with 0.2 NaOH, 10 min, 25°C, for antibody reprobing. Western blots were quantified using Adobe Photoshop software. In order to be able to compare all the samples per dietary condition for each antibody it was necessary to run two gels per antibody probe. To minimize any variability across gels, control and high fat diet samples were resolved and transferred for individual antibody comparisons at the same time in the same gel running/transfer apparatus. The blots were incubated in the same antibody solution and visualized and quantified at the same time. This minimized the variability of across-gel comparisons. Optical density of bands were normalized against their respective loading controls and averaged (+/−SD).

### Immunohistochemistry

After 22 weeks the animals were perfused with PBS containing CaCl_2_ and brain, hippocampus, visceral (pericardial/heart) fat and subcutaneous (abdominal/ stomach) fat was collected and immersion fixed for 24 hrs in 4% paraformaldehyde, cryoprotected through two successive 30% sucrose changes. Brains were embedded in gelatin and serially sectioned (40 µm) via freezing microtome and immunostained using anti-APP, β-amyloid, CD68, iNOS, Cox-2, or GFAP antibodies or respective secondary only antibodies. Subcutaneous and visceral fat tissue samples were serially cryosectioned (10 µm) or paraffin embedded and sectioned (7 µm) for immunostaining with anti-APP, β-amyloid, CD45 or CD68 antibodies or respective secondary only antibodies. Antibody binding in brain or fat was visualized using Vector VIP or DAB as chromogens (Vector Laboratories, Burlingame, CA, USA). For double labeling, immunostained tissue was stripped of antibodies with 0.2N HCl for 10 min prior to processing with the second primary antibody incubation and visualization steps. Images were taken using an upright Leica DM1000 microscope and Leica DF320 digital camera system. Figures were made using Adobe Photoshop 7.0 software.

### Macrophage Isolation and Stimulation

At 22 weeks non-elicited peritoneal macrophage were rinsed from the peritoneal cavity with sterile PBS and allowed to adhere to tissue culture wells for 2–3 hours in DMEM/F12 with 10% FBS and 5% horse serum. Nonadherent and non-macrophage cells were rinsed from the wells using ice cold DMEM/F12. The remaining macrophage were scraped from the wells and replated for stimulations.

### Microglia Isolation and Stimulation

Acutely isolated microglia were collected at 22 weeks as previously described [35]. Briefly, cortices were isolated, finely minced and filtered through 140 and 70 um filters then gently digested with DNAse I and collagenase before being separated on a Percoll gradient. The microglial layer was collected, counted and used for stimulations.

### Enzyme-linked Immunosorbent Assay

Isolated microglia and macrophage were plated (40,000 cell/150 µL) in DMEM/F12 overnight then media was collected for quantifying secreted TNF-α according to the manufacturer's protocol (R&D Systems, Minneapolis, MN, USA).

### Prostaglandin Analysis

PG were analyzed as previously described [101]. Brain samples (were homogenized in 1 mL of saline and extracted with 2 mL of acetone containing 0.005% butylated hydroxytoluene (BHT) following with 2 mL of chloroform with 0.005% BHT. PGE_2_d_4_ was used as internal standard. After extraction, the samples were dried under a stream of nitrogen and then redissolved in 30 µL of acetonitrile:water (1∶2 by volume). Reverse-phase LC electrospray ionization mass spectrometry was used for PG analysis. The PG were separated on a Luna C-18(2) (3 µm column, 100 A pore diameter, 150 x 2.0 mm) (Phenomenex, Torrance, CA, USA) with security guard cartridge system (Phenomenex, Torrance, CA, USA). The LC system consisted of an Agilent 1100 series LC pump with a wellplate autosampler (Agilent Technologies, Santa Clara, CA). The solvent system was composed of 0.1% formic acid in water (solvent A) and 0.1% formic acid in acetonitrile (solvent B). The flow rate was 0.2 mL/min. The separation program started with 10% of solvent B. At 2 min, the percentage of B was increased to 65% over 8 min, at 15 min the percentage of B was increased to 90% over 5 min, and at 35 min it was reduced to 10% over 2 min. Equilibration time between runs was 13 min. Mass spectrometry analysis was performed using a quadrapole mass spectrometer (API3000, Applied Biosystem, Foster City, CA, USA) equipped with a TurboIonSpray ionization source. Analyst software version 1.5.1 (Applied Biosystem, Foster City, CA, USA) was used for instrument control, data acquisition, and data analysis. The mass spectrometer was optimized in the multiple reaction-monitoring mode. The source was operated in negative ion electrospray mode at 450°C, electrospray voltage was −4250 V, nebulizer gas was 8 L/min and curtain gas was 11 L/min. Declustering potential, focusing potential, and entrance potential were optimized individually for each analyte. The quadrupole mass spectrometer was operated at unit resolution.

### Antibody-based Cytokine Array

At 7 months of age non-elicited peritoneal macrophage were rinsed from the peritoneal cavity with sterile PBS, counted, plated at 1.5×10^6^cells/well/6well dish and allowed to adhere to the tissue culture wells for 2–3 hours in DMEM/F12. Nonadherent and non-macrophage cells were rinsed from the wells using ice-cold DMEM/F12. Media containing isotype negative control 1 µg/mL IgG_1_ (Millipore, Billerica, Massachusetts USA) or APP agonist antibody 1 µg/mL 22C11 (Millipore, Billerica, Massachusetts USA) was added to the wells overnight for stimulation. The media was removed and used according to the manufacturer's protocol (Ray Biotech, Norcross, GA, USA) for the cytokine arrays. 5 animals per stimulation were analyzed. Optical density of dots were normalized against their respective positive controls and averaged (+/-SD).

### Adipocyte Isolation and Stimulation

Adipocyte cultures were generated via a modification of a prior method [102]. Subcutaneous abdominal adipose tissue was removed from the mice, rinsed with sterile Hanks Balanced Salt Solution (HBSS), minced with scissors, placed in 35 mm plates and incubated for 1 hr @ 37C on a rocker in a sterile enzyme solution (5mM glucose, 1.5% BSA, 5 mg collagenase in HBSS). Following incubation, adipocytes were spun at 186xg for 2 min and the lower layer was removed and discarded. The upper layer was resuspened in sterile HBSS to repeat the spin, rinse was repeated two times. The final upper layer was resuspended in DMEM/F12 + 10% FBS + 5% horse serum + antibiotics (0.05 mg penicillin/0.05 mg streptomycin/0.01mg neomycin/mL). The cells were added to 96 well plates, with DMEM/F12 + 10% FBS + 5% horse serum + antibiotics. The plates were then completely filled to the brim with media, covered with sterile parafilm, and inverted for 1 week to allow floating adipocytes to adhere to the tissue culture treated component of the plastic wells. After 1 week, the media was removed and the plates place right-side up so that adipocytes were now available for stimulation. The cells were treated with serum free DMEM/F12 and unstimulated (control) or stimulated for 24 hr with 1 µg/mL IgG_1_ (isotype negative control) or 1 µg/mL 22C11 (APP agonist antibody). After 24 hours, secreted LDH was measured according to the manufacture protocol for both the media and the cell lysates using commercial reagents (Promega, Madison, WI). Secreted media LDH values were normalized against cellular LDH values. Media was also collected for ELISA analysis quantifying secreted TNF-α according to the manufacturer's protocol (R&D Systems, Minneapolis, MN). Cells were also fixed, stained with Oil Red O (Alfa Aesar, Ward Hill, MN, USA) and DAPI (4′,6-diamidino-2-phenylindole) (Sigma Aldrich, St. Louis, MO, USA) counterstained and changes in Oil Red O absorbance (510 nm) normalized to DAPI absorbance (358/461 nm) were quantified via plate reader. Experiments were repeated three independent times.

### Statistical Analysis

The data were analyzed by unpaired two-tailed t-test with or without Welch correction for unequal variance as required. When three groups were analyzed one-way ANOVA with Holm-Sidak post hoc test or a Kruskal-Wallis nonparametric ANOVA with a Dunn's post hoc test were utilized for analysis.
